# Effects of Combined Plyometric and Short Sprints Training on Athletic Performance of Male U19 Soccer Players

**DOI:** 10.3389/fpsyg.2021.714016

**Published:** 2021-09-15

**Authors:** Ghaith Aloui, Hermassi Souhail, Lawrence D. Hayes, El Ghali Bouhafs, Mohamed Souhaiel Chelly, René Schwesig

**Affiliations:** ^1^Research Unit (UR17JS01) “Sport Performance, Health & Society”, Higher Institute of Sport and Physical Education of Ksar Saîd, University of La Manouba, Tunis, Tunisia; ^2^Physical Education Department, College of Education, Qatar University, Doha, Qatar; ^3^School of Health and Life Sciences, University of the West of Scotland, Glasgow, United Kingdom; ^4^Department of Sports Science, Martin-Luther-University Halle-Wittenberg, Halle (Saale), Germany; ^5^Department of Orthopaedic and Trauma Surgery, Martin-Luther-University Halle-Wittenberg, Halle (Saale), Germany

**Keywords:** stretch-shortening cycle, sprint training, plyometric training, team sports, soccer training, periodization

## Abstract

This project investigated adding 8 weeks of biweekly plyometric and short sprints training into standard training in elite youth soccer players. An experimental group (EG, *n* = 18, age: 17.6 ± 0.6 years, body mass: 67.6 ± 5.8 kg, height: 1.75 ± 0.06 m, and body fat: 11.5 ± 1.6%) and control group (CG, *n* = 18, age: 17.5 ± 0.6 years, body mass: 68.8 ± 3.6 kg, height: 1.77 ± 0.04 m, and body fat: 11.7 ± 1.2%) participated. Pre-intervention and post-intervention measures were squat-jump (SJ), countermovement-jump (CMJ), standing long jump (SLJ), 5 and 20 m sprints, change-of-direction ability (4 × 5 m sprint test [S 4 × 5 m] and sprint 9-3-6-3-9 m with backward and forward running [SBF]), repeated change of direction (RCOD), and static balance (the stork balance test). For all parameters, significant (*p* < 0.001, $\eta_{\text{p}}^{2}$ > 0.10) time and interaction (group × time) effects were observed. For three parameters (SBF, RCOD fastest time, and SLJ) no significant group effects were observed. The EG consistently showed a significantly higher performance level than the CG and a higher amount of effect sizes *d* (EG: *d*_range_: 1.27–2.61; CG: *d*_range_: 0.13–0.79) as an indicator for the development of performance between pre-intervention and post-intervention measures. Adding biweekly plyometric and short sprint training to standard training improves the athletic performance of young soccer players. Such plyometric and short sprint training conditioning can be highly recommended as part of the annual short training program for male elite under-19 (U19) soccer players.

## Introduction

Soccer is the most popular sport in the world and is played by men and women, young and adults with varying levels of expertise (Stølen et al., 2005). Soccer is an intermittent high-intensity sport that requires both physical and technical abilities during the gameplay (Stølen et al., 2005). In modern soccer, physiological and physical requirements are essential for optimum performance at all levels (Sáez de Villarreal et al., 2015). The ability of soccer players to repeatedly sprint with short recovery between sprints is called repeated sprint ability (RSA) (Girard et al., 2011) and has been considered an important variable of the performance in soccer (Rampinini et al., 2007). Rampinini et al. (2007) reported a large significant inverse association observed between RSA meantime and very high-intensity running performance in professional soccer players. Despite the relevance of sprint, change-of-direction, jump, and RSA to soccer performance, regular soccer training alone does not appear to improve these variables (Ramírez-Campillo et al., 2015; Rey et al., 2017). Therefore, it seems that the specificity of sports training, such as protocols targeting jumping, sprinting, changing direction, and RSA, is warranted to improve these capabilities (Peterson et al., 2006; Asadi et al., 2016).

Specific plyometric and short sprints training protocols were used to supplement the regular soccer training to improve the powerful actions such as sprinting and jumping performance associated with soccer performance (Venturelli et al., 2008; Mujika et al., 2009; Rumpf et al., 2016; Beato et al., 2018; Makaruk et al., 2020; Ramirez-Campillo and Sanchez-Sanchez, 2020; Sánchez et al., 2020). Knowing that a recent systematic review (Bedoya et al., 2015) reported that with the plyometric training programs, there is no more risk of injury than any other physical or recreational activity in which children and adolescents usually participate. The sprint training comprises maximum races varying distances, with acceleration-focused programs with shorter sprints (i.e., 20 m or less) (Spinks et al., 2007; Lockie et al., 2012, 2014). Recent studies (Rumpf et al., 2016; Pavillon et al., 2021) have reported significant improvements in athletic performance in young and adult athletes following the sprint-training protocol, but no injuries have been observed after this type of training. Plyometric training can put even more emphasis on the production of the stance force (Wallace et al., 2010), with exercises such as bounding and hopping producing much greater ground reaction forces compared to maximal sprinting (Mero and Komi, 1994).

Furthermore, a common trend in the training programs indicates that a combination of methods is most effective in improving performance than standalone approaches (Adams et al., 1992). Indeed, previous recent studies have reported improvement in athletic performance after combined plyometric and short sprint training in soccer players (Sáez de Villarreal et al., 2015; Hammami et al., 2020; Kargarfard et al., 2020). Recently, Hammami et al. (2020) reported such training-enhanced explosive actions, such as jumping, sprinting, and change-of-direction ability, in male soccer players (under-17, U17). Furthermore, Kargarfard et al. (2020) studied the effects of combined plyometric and short sprints in male under-19 (U19) soccer players. These authors reported significant improvement in sprint performance and change-of-direction ability, but no significant increase in RSA.

Few of the studies have investigated the effects of 8 weeks of biweekly plyometric and short sprints training on standard training in U19 soccer players. Despite the efficacious nature of plyometric training and short sprint training to enhance athletic performance, scarce research has focused on the effects of combined plyometric and short sprint training on jumping, sprinting, change of direction ability, RCOD, and balance in elite youth soccer players (U19). A reason could be the expected increased likelihood of injury combined with such type of high-intensity training. The aim of this study was therefore to evaluate the effects of replacing normal training during the season with combined plyometric and short sprints training in elite male junior soccer players (U19).

We hypothesized that replacing part of the regular in-season training with 8 weeks of biweekly combined plyometric and short sprints training would improve both horizontal and vertical jump performance, sprint performance, change of direction ability, and balance when compared with the control group (CG), who maintained their habitual training during the season training. Specifically, we examined vertical and horizontal jump performance (i.e., squat jump [SJ], countermovement jump [CMJ], and the standing long jump [SJL]), sprint performance (i.e., 5 and 20 m sprints), change-of-direction ability (4 × 5 m sprint test [S 4 m × 5 m] and sprint 9-3-6-3-9 m with backward and forward running [SBF]), repeated change of direction (RCOD) ability, and static balance performance (the stork balance test).

## Materials and Methods

### Participants

The investigation was completed in an in-local soccer-season period, of 2020/2021. The procedures were approved by the national university institutional review board (approval number: KS00000-KS2021 and date of approval December 10, 2020) for human subjects and complied with the requirements of the Declaration of Helsinki. Participants (and their guardians, in the case of minors) provided written informed consent.

Thirty-six players from a single male soccer team competing in the first national division participated. They had a playing experience of 7.7 ± 0.8 years and before the study, they were examined by the team doctor. Participants were randomly allocated between an experimental group (EG, *n* = 18, age: 17.6 ± 0.56 years, body mass: 67.6 ± 5.83 kg, height: 1.75 ± 0.06 m, and body fat: 11.5 ± 1.61%) and a CG (*n* = 18, age: 17.5 ± 0.55 years, body mass: 68.8 ± 3.59 kg, height: 1.77 ± 0.04 m, and body fat: 11.7 ± 1.15%). No significant initial intergroup differences of anthropometric characteristics and age were observed (*p* > 0.40, $\eta_{\text{p}}^{2}$ < 0.04).

Before the study, participants were well-conditioned as they had completed an 8-week training period of five to six training sessions per week. These 8 weeks were comprised of a resistance training program initially targeting hypertrophy and muscle force. In the final 3 of these 8 weeks, muscular power was targeting by reducing the load and increasing the contraction velocity. Every weekend participants partook in a non-competitive training match. Participants continued to participate in five sessions per week of training when the championship season had begun.

### Experimental Design

Participants did not train in their own time and therefore only trained with the soccer team during the study. Both the EG and CG trained five times per week (~90 min per session), with a competitive match at the weekend. Conditioning sessions were undertaken two times per week. The first of these sessions developing aerobic fitness through high-intensity interval training and small-sided games (Karahan, 2020). The second session comprised resistance training exercises such as half squats, overhead lunges, and CMJ and SJs. The other training sessions sought to develop tactical technical skills (Table 1).

**Table 1:** Details of general training routine during the week performed by both control and experimental groups over the 8-week intervention.

**Days**	**Objectives**
Mondays	Rest
Tuesdays	Aerobic training and defensive tactics training
Wednesdays	Maximum power, aerobic training and defensive tactics training
Thursdays	Power anaerobic training and defensive and offensive tactics training
Fridays	Technical training and offensive tactics training
Saturdays	Technical training and offensive tactics training
Sunday	Official games

**Figure 1:**
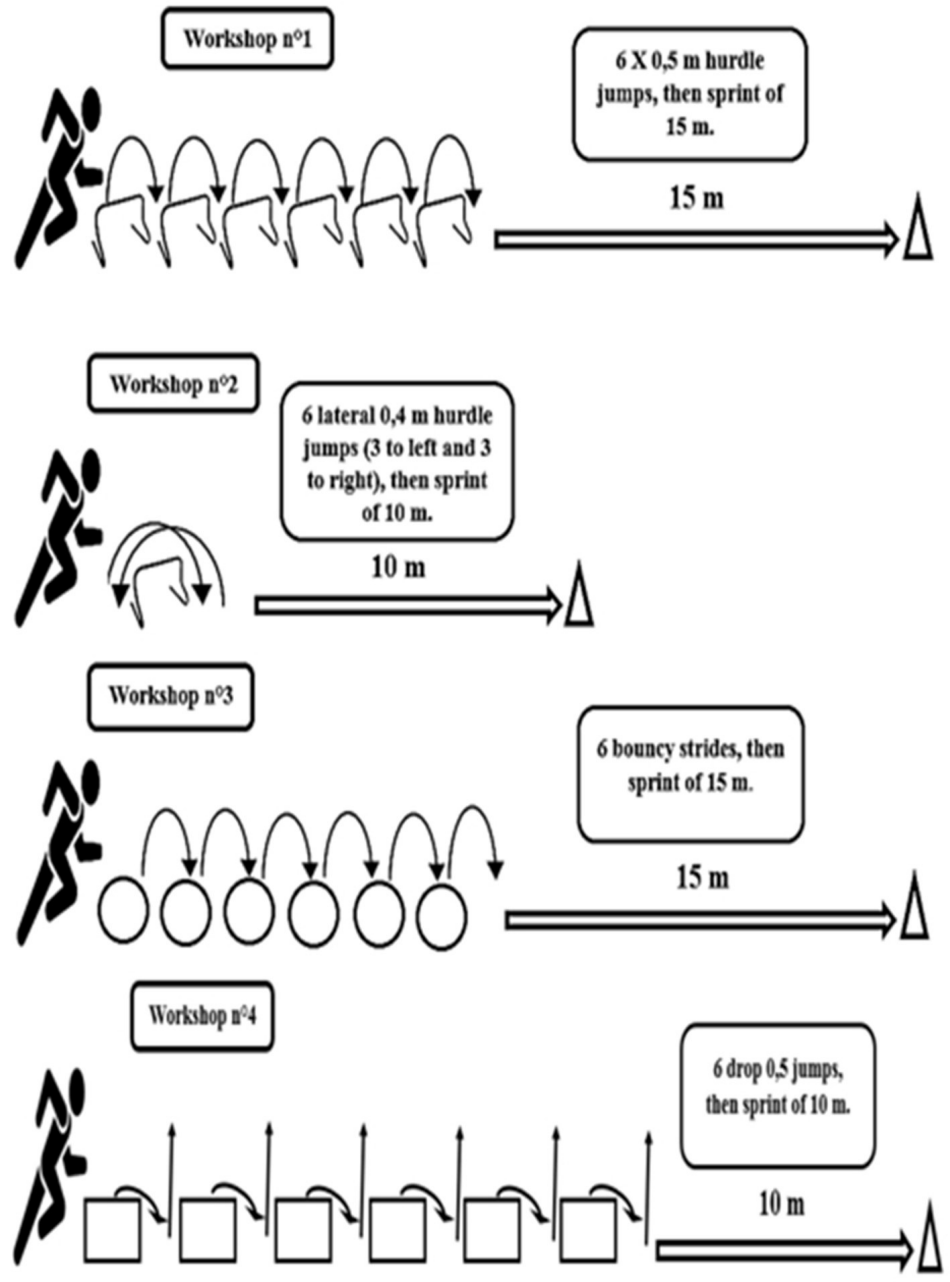
Exercise used in loaded combined plyometric and short sprint training jump.

The CG maintained their normal training schedule throughout the 8-week intervention, whereas every Tuesday and Thursday the EG replaced the technical-tactical part of their standard regimen by combined plyometric and short sprints training. No injuries were incurred for the CG and EG during the 8-week intervention. Two familiarization sessions were held 2 weeks before definitive testing, which began 2 months into the competitive season. The combined plyometrics and short sprints training program consisted of principal workshops, two times per week (Figure 1).

Each workshop began with plyometric exercises (hurdle jumps, lateral hurdle jumps, bouncy strides, and drop jumps) and finished with a short sprint. Details are given in Table 2.

**Table 2:** Plyometric components were introduced into the program of the experimental group.

**Week**	**Workshop 1**	**Workshop 2**	**Workshop 3**	**Workshop 4**	**Total (contact)**
**1**	3 Repetitions	3 Repetitions	3 Repetitions	3 Repetitions	72
**2**	3 Repetitions	3 Repetitions	3 Repetitions	3 Repetitions	72
**3**	4 Repetitions	4 Repetitions	4 Repetitions	4 Repetitions	96
**4**	4 Repetitions	4 Repetitions	4 Repetitions	4 Repetitions	96
**5**	5 Repetitions	5 Repetitions	5 Repetitions	5 Repetitions	120
**6**	5 Repetitions	5 Repetitions	5 Repetitions	5 Repetitions	120
**7**	6 Repetitions	6 Repetitions	6 Repetitions	6 Repetitions	144
**8**	6 Repetitions	6 Repetitions	6 Repetitions	6 Repetitions	144

The plyometric training protocol was based on previously published recommendations for training volume and intensity from Bedoya et al. (2015). Furthermore, the sprint training protocol was based on previously published recommendations for sprint distance (Sáez de Villarreal et al., 2015; Kargarfard et al., 2020). During the whole intervention period (8 weeks), we did not observe any injuries.

### Testing Schedule

Two familiarization sessions were conducted 2 weeks before testing and 2 months after commencement the competitive season had started. Tests were conducted at least 3 days after the last competitive match and 5–9 days after the previous training session. Testing took place on a tartan surface integrated into the weekly training schedule of players and a standardized warm-up preceded each test. Tests were completed over three separate testing days in the following order: anthropometric assessment, SJ, CMJ, and 4 × 5 m sprint test (S 4 × 5 m) (all day 1); the stork balance test, 5 and 20 m sprints, and SBF (all day 2); and the SLJ and the RCOD (both day 3). The SJ, CMJ, 4 × 5 m, the stork balance test, 5 and 20 m sprints performance, SBF, SLJ, and the RCOD have all been previously described in detail (Negra et al., 2019; Hammami et al., 2020) so are not outlined here for brevity and to avoid self-plagiarism.

### Statistical Analysis

Statistical analysis was performed using SPSS version 25.0 for Windows (SPSS Inc., IBM, Armonk, NY, USA). Data were tested for normal distribution (the Shapiro-Wilk test) and homogeneity of variance (Levene's test for equality of variances).

Mean differences of anthropometric and performance parameters between groups (EG vs. CG) and time (test vs. retest) were tested using a univariate, two-factor (time and group) general linear model (Bortz, 1999). Differences between means were considered meaningful if *p* < 0.05 and partial eta-squared ($\eta_{\text{p}}^{2}$) > 0.15 (Richardson, 2011; Hurlbert et al., 2019). A power calculation (nQuery Advisor 4.0; Statistical Solutions, Saugus, MA, USA) was performed using previous data (Hermassi et al., 2021), which suggested a sample size of 13 participants in each group, where the study would have 80% power to detect a mean difference of 2.90 cm in CMJ performance using a two-sided *t*-test with a significance level of *p* < 0.05 under the assumption of a pooled standard deviation of 2.50 cm (Bortz, 1999). Due to the relatively small number of cases in each age group (*n* = 18) and to avoid an overestimation of mean differences, the interpretation of results was primarily based on $\eta_{\text{p}}^{2}$.

The effect size (*d*) (the mean difference of scores divided by the pooled SD) was calculated for each parameter (Hartmann et al., 1992), as is interpreted as trivial (<0.20), small (≥0.20–0.49), moderate (≥0.50–0.79), and large (≥0.80) (Cohen, 1988).

Pearson product-moment correlation coefficients (*r*) were interpreted as follows: <0.1, trivial; 0.1–0.3, small; 0.3–05, moderate; 0.5–0.7, large; 0.7–0.9, very large; and 0.9–1.0, almost perfect (Cohen, 1988). Therefore, *r*^2^ > 0.5 (explained variance > 50%) was defined as meaningful and is marked in bold. Regarding the sample size of *n* = 36, the critical value for the product-moment correlation based on a two-sided *t*-test and α = 5% is *r* = 0.325 (Willimczik, 1997).

Reliability was assessed using intraclass correlation coefficients (Vincent, 1995) and the coefficients of variation (CV) over consecutive pairs of intra-participant trials (Schabort et al., 1998). All measures of vertical and horizontal jumping, sprinting, change of direction ability, and balance performance had an intraclass correlation coefficient (ICC) > 0.80 and a CV < 5%. Data are reported as mean ± SD. The interpretation of the reliability values was based upon Shrout and Fleiss (1979), Cohen (1988), Hopkins (2000), and Portney and Watkins (2009). The ICC was considered to show excellent relative reliability (intersubject variability) if it was >0.75, 0.40–0.75 was considered fair to good, and <0.40 was considered poor. The CV (the indicator of measurement variability and random error, i.e., absolute reliability) can be defined as good with values <10% (Cormack et al., 2008; Brughelli and Van Leemputte, 2013).

## Results

### Reliability

ICCs, the CIs, and CV assessing the reliability for vertical and horizontal jump, sprint times, change of direction, and balance tests are summarized in Table 3.

**Table 3:** Interclass correlation coefficient (ICC, 95% CI) and coefficient of variation (CV) showing acceptable reliability for all performance.

	**ICC (95% CI)**	**CV (95%CI) [%]**
**Sprint times**		
5 m (s)	0.95 (0.91–0.97)	1.8 (1.4–2.2)
20 m (s)	0.96 (0.92–0.98)	1.5 (1.3–1.8)
**Change of direction test**		
Sprint 4 x 5 m (s)	0.93 (0.90–0.96)	1.2 (1.1–1.3)
Sprint 9-3-6-3-9 m With Backward and Forward Running (s)	0.95 (0.91–0.98)	1.1 (1.1–1.2)
**Vertical jump**		
SJ (cm)	0.96 (0.92–0.98)	2.1 (1.8–2.5)
CMJ (cm)	0.97 (0.94–0.99)	2.3 (2.0–2.8)
**Horizontal jump**		
SLJ (cm)	0.96 (0.93–0.98)	3.2 (2.5–4.0)
**Stork balance test**		
Right leg (s)	0.84 (0.69–0.90)	3.7 (2.8–4.8)
Left leg (s)	0.82 (0.67–0.88)	3.8 (2.7–4.8)

ICC values ranged from 0.82 (the stork balance test, left leg) to 0.97 (CMJ). All CV moved below the threshold of 5%. The lowest (1.1%) and the highest (3.8%) values were calculated for RCOD and stork balance test, left leg.

### Normal Distribution and Variance Homogeneity

The variables BMI (*p* = 0.036), SJ (*p* = 0.028), sprint 20 m (*p* = 0.002), and stork balance test (*p* = 0.002) were not normally distributed. Regarding variance homogeneity, four parameters (SJ: *p* = 0.024; RCOD_best_: *p* = 0.027; RCOD_mean_: *p* = 0.008, and BMI: *p* = 0.010) were not homogenous in variance.

### Effect of Training on Jump and Sprint Performance

For sprint, vertical and horizontal jump performance and intervention effects (group × time interaction) were significant for all parameters and differed between $\eta_{p}^{2}$ = 0.917 (sprint 5 m) and $\eta_{p}^{2}$ = 0.968 (CMJ) (Table 4).

**Table 4:** Jump and sprint test performances in experimental and control groups before and after the 8-week intervention.

	**Experimental (n = 18)**			**Control (n = 18)**			**Variance Analysis*****p*** ($\eta_{p}^{2}$)		
	**Test**	**Retest**	**d**	**Test**	**Retest**	**d**	**group**	**time**	**group × time**
**Vertical jump**									
SJ (cm)	29.8 ± 3.80	35.5 ± 3.63	**1.53**	29.5 ± 2.42	30.6 ± 2.36	0.46	**0.016 (0.160)**	**<0.001 (0.983)**	**<0.001 (0.964)**
CMJ (cm)	32.3 ± 3.84	38.3 ± 3.66	**1.60**	32.1 ± 2.96	33.4 ± 2.86	0.79	0.029 (0.133)	**<0.001 (0.986)**	**<0.001 (0.968)**
**Horizontal jump**									
SLJ (m)	2.05 ± 0.22	2.33 ± 0.22	**1.27**	2.05 ± 0.15	2.11 ± 0.14	0.41	0.091 (0.082)	**<0.001 (0.976)**	**<0.001 (0.940)**
**Sprin**t									
5 m (s)	0.94 ± 0.09	0.83 ± 0.07	**1.38**	0.96 ± 0.09	0.94 ± 0.09	0.22	0.032 (0.128)	**<0.001 (0.959)**	**<0.001 (0.917)**
20 m (s)	3.19 ± 0.24	2.88 ± 0.21	**1.38**	3.27 ± 0.18	3.20 ± 0.17	0.17	**0.004 (0.214)**	**<0.001 (0.970)**	**<0.001 (0.930)**

*Meaningful effects (criteria: p < 0.05 and $\eta_{p}^{2}$ > 0.15) are highlighted in bold*.

The effect sizes (*d*) moved on a markedly higher level for EG (*d*_range_: 1.27–1.60) than for CG (*d*_range_: 0.17–0.79). SLJ (*p* = 0.091, $\eta_{p}^{2}$ = 0.082), CMJ (*p* = 0.029, $\eta_{p}^{2}$ = 0.133), and sprint 5 m (*p* = 0.032, $\eta_{p}^{2}$ = 0.128) were the parameters with non-significant group effects as an indicator for similar conditions before the intervention.

### Effect of Training on Change of Direction Ability and Balance

The S 4 × 5 m and the SBF showed relevant changes over the time for the EG (*d* = 1.91 and 1.46) in contrast to the CG (*d* = 0.70 and 0.51) (Table 5).

**Table 5:** Agility and balance test performances in experimental and control groups before and after the 8-week intervention.

	**Experimental (n = 18)**			**Control (n = 18)**			**Variance Analysis*****p*** ($\eta_{p}^{2}$)		
	**Test**	**Retest**	**d**	**Test**	**Retest**	**d**	**group**	**time**	**group × time**
**Change of direction Performance**									
Sprint 4 x 5 m (s)	6.12 ± 0.36	5.49 ± 0.30	**1.91**	6.15 ± 0.25	5.99 ± 0.21	0.70	**0.009 (0.183)**	**<0.001 (0.980)**	**<0.001 (0.947)**
Sprint 9-3-6-3-9 m with backward and forward running (s)	8.20 ± 0.52	7.50 ± 0.44	**1.46**	8.16 ± 0.40	7.96 ± 0.35	0.51	0.156 (0.058)	**<0.001 (0.965)**	**<0.001 (0.898)**
**Repeated Change of Direction Ability parameters**									
Fastest time (s)	6.53 ± 0.35	5.95 ± 0.32	**1.73**	6.49 ± 0.27	6.37 ± 0.25	0.46	0.065 (0.097)	**<0.001 (0.983)**	**<0.001 (0.962)**
Mean time (s)	6.63 ± 0.36	6.05 ± 0.33	**1.68**	6.61 ± 0.26	6.49 ± 0.24	0.48	0.046 (0.112)	**<0.001 (0.982)**	**<0.001 (0.959)**
**Stork balance test**									
Right leg (s)	13.5 ± 4.69	22.7 ± 6.95	**1.58**	13.3 ± 5.33	14.0 ± 5.34	0.13	0.023 (0.143)	**<0.001 (0.902)**	**<0.001 (0.870)**
Left leg (s)	11.1 ± 2.39	19.0 ± 3.66	**2.61**	11.3 ± 3.41	12.1 ± 3.44	0.23	**0.004 (0.221)**	**<0.001 (0.951)**	**<0.001 (0.929)**

*Meaningful effects (criteria: p < 0.05 and $\eta_{p}^{2}$ > 0.15) are highlighted in bold*.

The same relation was found for RCOD (EG: *d* = 1.73 and 1.68 vs. CG: *d* = 0.46 and 0.48) and the stork balance test (EG: *d* = 1.58 and 2.61 vs. CG: *d* = 0.13 and 0.23). For all the parameters, significant interaction effects were observed as an indication of the effectiveness of the intervention. Partial eta squared ($\eta_{\text{p}}^{2}$) consistently moved on a very high level (from 0.870 to 0.962).

SBF (*p* = 0.156, $\eta_{p}^{2}$ = 0.058), RCOD_fastest_ _time_ (*p* = 0.065, $\eta_{p}^{2}$ = 0.097), RCOD_mean_ _time_ (*p* = 0.046, $\eta_{p}^{2}$ = 0.112), and the stork balance test right leg (*p* = 0.023, $\eta_{p}^{2}$ = 0.143) did not show a significant group difference before intervention.

### Relationships Between Anthropometric and Performance Parameters

Correlations of practical value (*r* ≥ 0.5) were not found between anthropometric and performance parameters (Table 5). The largest relevant relationship was detected for BMI and sprint 4 × 5 m (*r* = 0.486).

Between performance parameters from different categories (sprint, jump, change of direction, RCOD, and balance), the following relevant relationships were calculated:

- sprint 5 m/RCOD_mean_: *r* = 0.545;
- sprint 5 m/RCOD_best_: *r* = 0.558;
- SBF/RCOD_mean_: *r* = 0.521;
- SBF/RCOD_best_: *r* = 0.504;
- Sprint 4 × 5 m/RCOD_mean_: *r* = 0.502.

## Discussion

This study examined the effect of replacing part of the regular in-season training with 8 weeks of biweekly combined plyometric with short sprints training in elite adolescent soccer players. The present data show substantial gains in many measures of performance in the EG over the training period of combined plyometric and short sprints training. In this context, we accept our hypothesis that replacing part of the regular in-season training with 8 weeks of biweekly combined plyometric with short sprints training would improve both horizontal and vertical jump performance, sprint performance, and change of direction ability. The performance of the CG decreased in comparison with the training group, suggesting that although the training group received an effective training stimulus, the training load in the CG was not enough to increase the physical performance of male U19 soccer players.

### Effect of Training on Jump Performance

Explosive strength in the form of jumping was seen as a primary ability to optimize soccer performance and taken into account in fitness and talent screening tests (Castagna and Castellini, 2013). Arnason et al. (2004) reported that competitive high-level soccer players jump higher than lower-performance level players. The present results showed significant intervention effects, in the EG compared to the CG, on vertical and horizontal jump performance (Table 4). To the knowledge of the authors, our study remains the first that investigated the effect of combined plyometric and short sprints training on jump performance in youth soccer players (U19). This corroborated findings of Hammami et al. (2020), who studied the effects of the combination of 10-week plyometric training and short sprint exercises in male soccer players (U17) and noted improvement in vertical and horizontal jump performance. Moreover, Sáez de Villarreal et al. (2015) observed significant increases in horizontal jump performance following combined plyometric and short sprints training, in male soccer players (under-15, U15). The improvement in jump performance following plyometric training may be partially attributable to a change in the level of neuromuscular activation, motor intermuscular and intramuscular coordination, and elastic advantages of SSC and/or changes in muscle typology (Diallo et al., 2001; Markovic and Mikulic, 2010; El-Ashker et al., 2019).

### Effect of Training on Sprint Performance

In-game situations, player sprints on average between 110 and 330 m (Dalen et al., 2016), with the average distance of the sprint activity is 16.5 m (Carling et al., 2012). Jovanovic et al. (2011) reported that elite level soccer players run more high-intensity running, involves more short sprints, and have faster reactions than others at a lower level, during the game. The present results showed significant intervention effects, in the EG compared to the CG, on the sprint performance over distances of 5 and 20 m (Table 4). To the best of our knowledge, the present investigation is the first to have studied the effects of a combined plyometric and short sprint training programs on start speed, in youth soccer players (U19). Our data are following the existing literature which has reported improved sprint performance after several programs, namely, combined plyometric and short sprints (Sáez de Villarreal et al., 2015; Almoslim, 2016; Hammami et al., 2019b; Kargarfard et al., 2020), plyometric training (Hammami et al., 2019b; Ramirez-Campillo and Sanchez-Sanchez, 2020; Pardos-Mainer and Lozano, 2021), and sprint training (Venturelli et al., 2008; Mujika et al., 2009; Rumpf et al., 2016).

Recently, Kargarfard et al. (2020) studied the effects of the combination of 6-week plyometric and short sprints exercises in male U19 soccer players; reporting improved sprint performance over 30 m. Moreover, Hammami et al. (2020) observed significant increases in 5, 10, 20, 30, and 40 m sprint times following combined loaded and unloaded plyometric and short sprints training, in male U17 soccer players. Indeed, gains in sprint performance as a result of combined plyometric and short sprints training in male U15 soccer players have been observed (Sáez de Villarreal et al., 2015). Improvements in sprint performance occur mainly due to neural factors such as improved intermuscular coordination, increased excitability of the Hoffman reflex (H-reflex), and enhanced motor unit recruitment strategy (Aagaard et al., 2002; Herrero et al., 2006; Markovic et al., 2007; Thomas et al., 2009; Markovic and Mikulic, 2010; Wu et al., 2010; Faude et al., 2012). Moreover, Sáez de Villarreal et al. (2015) reported that improved sprint performance after combined plyometric and short sprints training may be related to the effect of a positive number of exercises to ensure sufficient performance of neuromuscular and metabolic systems of participants.

### Effect of Training on Change of Direction Ability

During a match, soccer players perform sprints with the change of direction movements every 2–4 s (Popowczak et al., 2019), in different directions and at different angles (Little and Williams, 2005; Stølen et al., 2005; Sporis et al., 2009; Comfort et al., 2014). The present results showed significant intervention effects, in the EG compared to the CG, on change of direction ability (Table 4). There are few studies evaluating sprint performance after combined plyometric and short sprints training programs on change of direction ability, in youth soccer players (U19) (Kargarfard et al., 2020). We report the improved change of direction ability herein, which corroborates the results of previous recent studies (Sáez de Villarreal et al., 2015; Hammami et al., 2020; Kargarfard et al., 2020), following combined plyometric and short sprints training in male youth soccer players. However, our results contrast with the results of Beato et al. (2018) who reported no gain in change-of-direction ability after combined plyometric and sprint with change-of-direction training in youth soccer players (U18). Differences in training protocol, intervention period, and characteristics of participants could contribute to divergences between studies. The mechanism responsible for the improvement of the ability to change direction in our study may be associated with neural adaptations and improved motor unit recruitments (Aagaard et al., 2002). Additionally, improvements in change-of-direction ability can also be attributed to mechanical factors such as improved rate of force development (Miller et al., 2006; Sheppard and Young, 2006), and in movement efficiency (Young et al., 1995), caused by SSC exercises such as plyometric implemented in our study.

### Effect of Training on Repeated Change of Direction Ability

It has been shown that RSA is a significant factor in determining success in soccer (Rampinini et al., 2007; Impellizzeri et al., 2008). Rampinini et al. (2007) reported significant correlations between running distances covered during the actual matches and mean sprint times on an RSA test, in professional soccer players. The present results showed significant intervention effects, in the EG compared to the CG, on-call RCOD parameters except for the fatigue index (Table 4). To the best of our knowledge, the present investigation is the first to have studied the effects of combined plyometric and short sprints on RCOD ability, in young soccer players (U19). Our results are confirmed by Hammami et al. (2020) who observed improvement in RCOD following combined plyometric and short sprints training in male U17 soccer players. In contrast, our results contradict those of Kargarfard et al. (2020) who reported no improvement in RSA following combined plyometric and short sprints training in male U19 soccer players. Improvement in RCOD ability could be explained by increased efficiency in running (Balsalobre-Fernández et al., 2016) or an enhanced tendon stiffness (Markovic and Mikulic, 2010) which allows for a faster transfer of force from the contracting muscles, reducing reaction times and improving the ability to change direction (Markovic and Mikulic, 2010; Balsalobre-Fernández et al., 2016).

### Effect of Training on Balance Performance

Balance in soccer is necessary to maintain a one-leg posture while precision shooting, dribbling, and passing the ball (Paillard et al., 2006; Greg, 2009). Tropp et al. (1984) have shown that soccer players with functional ankle instability and low balance have a significantly increased risk of ankle sprain recurrence. The present results showed significant intervention effects, in the EG compared to the CG, on static balance performance for both legs (Table 4). Our study seems the first to have examined the effects of the combined plyometric and short sprints training programs on balance performance, in young soccer players (U19). Indeed, the EG in this study improved static balance compared to the CG which corroborates data of Hammami et al. (2020) who reported improves static and dynamic balance performance as a result of an 8-week plyometric and short sprints exercise program in male soccer players (U17). Makhlouf et al. (2018) also reported increased static and dynamic balance performance following combined plyometric and short sprint with change-of-direction training in prepubertal male soccer players. Contrary to the gains in static balance performance noted in this study, Hammami et al. (2019a) showed no significant effects in this skill, following combined plyometric and short sprint with change-of-direction training in male U15 handball players. The improvement observed of balance performance could be due to either an improved motor coordination of the lower extremity muscles (Trecroci et al., 2015; Hammami et al., 2017) and improvement in proprioception and neuromuscular control (Michailidis et al., 2019).

### Limitations

Our observations to date are mainly applicable to a category of elite adolescent soccer players. The age range of participants in the present study varied between 16 and 18 years. Although sexual maturation of groups was not considered in this study, future studies should include an assessment of sexual maturation when examining the effects of combined plyometric and short sprint training on the athletic performance of youth soccer players. In addition, it would be advantageous for further studies to include laboratory tests of fitness relative to the studied population, rather than field tests.

Furthermore, future studies should extend these observations to other disciplines, age groups, gender, and other competitive levels. It is also necessary to compare the gains in test performance with the analysis of performance during competitions to determine whether better fitness translates into improved match performance. This aspect, that the analysis was not conducted in competition match level (as opposed to training level) limited the presented study and should be addressed in further investigations.

Furthermore, neuromuscular mechanisms that underlie the improvements reported here can also be an area of future research. Finally, given that starting speed is a vital ability for soccer players, it is necessary to ask whether an increase in the intensity or volume of combined plyometric and short sprints training could make the new diet that we evaluated more effective at improving this capacity.

### Practical Applications

The present study shows the practical value of substituting a part of the usual soccer training regimen of male adolescent players with 8 weeks of in-season combined plyometric and short sprint training. This program improves physical abilities such as jumping, sprinting, change-of-direction ability, RCOD, and balance may also reduce the risk of injuries of the lower limb. Therefore, it seems reasonable to incorporate this form of training into traditional soccer technical and tactical training during the season to augment physical fitness. This type of training does not require the commitment of resources and is simple to implement by a strength and conditioning coach.

## Conclusions

In conclusion, adding combined plyometric and short sprints training to standard training improved jump performance, sprinting, change-of-direction ability, the RCOD, and balance. Therefore, soccer coaches and practitioners should incorporate combined plyometric and short sprints training into soccer training to enhance specific and non-specific fitness. Most of the gains associated with combined plyometric and short sprints training seem of substantial size, and should thus be of interest for both soccer players and their coaches. We would also encourage further investigation of the many potential factors underlying the increased performance during combined plyometric training, short sprint, and tapering. Factors yet to be clarified include both the optimal intensity of effort during tapering and its duration.

## Data Availability Statement

The original contributions presented in the study are included in the article/supplementary material, further inquiries can be directed to the corresponding author/s.

## Ethics Statement

The studies involving human participants were reviewed and approved by The procedures were approved by the national university institutional review board (approval number: KS00000-KS2021 and date of approval 10th December 2020) for human subjects and complied with the requirements of the Declaration of Helsinki. Participants (and their guardians, in the case of minors) provided written informed consent. Written informed consent to participate in this study was provided by the participants' legal guardian/next of kin.

## Author Contributions

GA and MC: conceptualization. GA: methodology, software, formal analysis, investigation, data curation, writing (original draft preparation), and project administration. GA, MC, and HS: validation. MC: resources and supervision. HS and LH: writing (reviewing and editing). EB: visualization. RS: funding acquisition. All authors have read and agreed to the published version of the manuscript.

## Conflict of Interest

The authors declare that the research was conducted in the absence of any commercial or financial relationships that could be construed as a potential conflict of interest.

## Publisher's Note

All claims expressed in this article are solely those of the authors and do not necessarily represent those of their affiliated organizations, or those of the publisher, the editors and the reviewers. Any product that may be evaluated in this article, or claim that may be made by its manufacturer, is not guaranteed or endorsed by the publisher.
